# Semi-supervised gene shaving method for predicting low variation biological pathways from genome-wide data

**DOI:** 10.1186/1471-2105-10-S1-S54

**Published:** 2009-01-30

**Authors:** Dongxiao Zhu

**Affiliations:** 1 Department of Computer Science, University of New Orleans, New Orleans, LA 70148, USA; 2 Research Institute for Children, Children's Hospital, New Orleans, LA 70118, USA

## Abstract

**Background:**

The gene shaving algorithm and many other clustering algorithms identify gene clusters showing high variation across samples. However, gene expression in many signaling pathways show only modest and concordant changes that fail to be identified by these methods. The increasingly available signaling pathway prior knowledge provide new opportunity to solve this problem.

**Results:**

We propose an innovative semi-supervised gene clustering algorithm, where the original gene shaving algorithm was extended and generalized so that prior knowledge of signaling pathways can be incorporated. Different from other methods, our method identifies gene clusters showing concerted and modest expression variation as well as strong expression correlation. Using available pathway gene sets as prior knowledge, whether complete or incomplete, our algorithm is capable of forming tightly regulated gene clusters showing modest variation across samples. We demonstrate the advantages of our algorithm over the original gene shaving algorithm using two microarray data sets. The stability of the gene clusters was accessed using a jackknife approach.

**Conclusion:**

Our algorithm represents one of the first clustering algorithms that is particularly designed to identify signaling pathways of low and concordant gene expression variation. The discriminating power is achieved by manufacturing a principal component enriched by signaling pathways.

## Background

Gene clustering that assigns group membership(s) to each gene is a widespread pattern extraction technique. Genes sharing the same membership are often hypothesized to be regulated by the same defined or undefined genomic influence, such as cellular pathway. Model-free clustering techniques such as K-means and hierarchical clustering [1-3] are widely used. One limitation of these approaches, as pointed out by many researchers, e.g. [4], is that each gene can only belong to a single cluster. These types of gene clustering algorithms are thus called mutually exclusive clustering. In the context of cellular pathways, they assume that one gene can only be regulated by one pathway at a time, which apparently, is not the case. Model-based clustering or soft clustering [5-8] provides mechanisms to relax this stringent assumption by introducing "probabilistic" or "fuzzy" memberships. However, these "soft" memberships do not biologically account for the fact that one gene is often simultaneously regulated by multiple genomic influences.

Singular value decomposition (SVD) [9-11] has shown great promise towards deconvolving channels of genomic influence. Assuming rows of data matrix correspond to genes and columns correspond to physiological/genetic conditions under which the gene expression abundance was interrogated using gene chips, the SVD factors the data matrix into three matrices. The first matrix, which contains most of information, is called a gene coefficient matrix where each column (principal component, PC) defines a preliminary gene cluster that might be regulated by a specific genomic influence. We will describe more details of SVD in the method section. SVD has been repeatedly shown to be able to deconvolve the observed gene expression signal into a composite of multiple overlapping genomic influences, many of them correspond to signaling pathways [9,11].

Thus SVD provides a methodology base for non-mutually exclusive clustering. The gene clusters generated by SVD are often preliminary due to the fact that many non-relevant genes might contaminate the PC's that define gene clusters. Hastie et al [4] proposed removing non-relevant genes in an iterative fashion, in which the least correlated genes with the leading PC is treated as non-relevant. The gene shaving algorithm quickly became an important tool in the pattern discovery arsenal. It iteratively searches for clusters of genes showing high variation across the samples, and correlation across the genes [8]. The former is achieved by working with the leading PC and the latter is achieved by iteratively discarding non-relevant genes to the cluster. There are other types of non-mutually exclusive clustering methods as well, such as plaid model [12].

The underlying assumption of the gene shaving algorithm is that the leading PC accounting for the largest portion of variation is always of exclusive interest to the investigator [4,13]. Consequently the algorithm iteratively refines the first gene cluster defined by the first PC by shaving off a proportion of genes that are least correlated with the leading PC. The second gene cluster is formed by performing the same procedure on the orthogonal data, resulting from the residuals of regression, and so on. However, the underlying assumption that the whole algorithm is based on is not always true for every single case. In fact, gene expression in many signaling pathways show modest but concordant changes. The gene shaving algorithm would most likely to fail in these cases by working exclusively with the leading PC.

Gene set based methods, such as Gene Set Enrichment Analysis (GSEA) were designed to overcome this limitation. Since it's first introduction in 2003 [14], it has been widely applied to interpret genome-wide expression profiles [15,16]. However, the approach only ranks pre-compiled gene sets according to the relevancy to the data and does not predict any new genes in the gene sets. Therefore, it strictly depends on the availability and validity of *a priori *defined gene sets. In reality a gene set is not always available in a complete and accurate format. What is typically available is partial pathway learned from empirical experimental studies.

We seek a seamless combination of the strengths of the two methodological frameworks. We manufacture a PC that is most enriched by prior knowledge (signaling pathway of interest). Performing the analysis iteratively we will be able to identify the gene cluster showing modest but concordant changes. In many cases, we are further interested in finding genes that are concordantly up or down-regulated by genomic influences. Therefore, it might be beneficial to turn our attention not only to the PC that the prior knowledge is most enriched, but also to the positive PC and the negative PC separatively. The hypothesis can be substantiated by previous works that positive and negative PC's can be enriched by completely different biological functions, e.g. [11].

**Figure 1:**
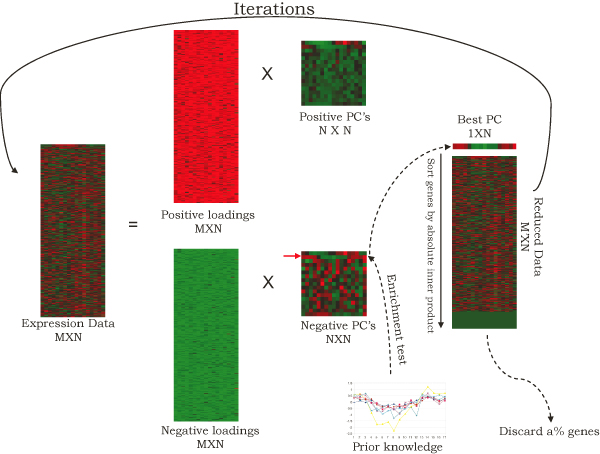
**The schematic diagram of the proposed algorithm**. "Enrichment test" means to determine the PC(s) that are most enriched by a prior knowledge gene set. *α*% is set to 10% following Hastie et al [4].

In our work, we eliminate non-relevant genes iteratively following and improving the procedure used in the gene shaving algorithm [4]. In each iteration, a weighted average expression profile was calculated and used as the seed profile to rank genes. With the heuristic removal of non-relevant genes at the beginning of the iterations, and some relevant genes by the end, the enrichment of prior knowledge has seen a sharp increase, followed by a gradual decrease. We then propose a trace-back step to retrieve the gene cluster in which enrichment of prior knowledge is maximized (Figure 1).

## Results

We aim to demonstrate that the proposed algorithm is capable of identifying tightly regulated gene sets showing modest and concerted variation using incomplete prior knowledge and real-world microarray data set. Ground truth, which indicates a "complete" gene set used as precondition for applying GSEA algorithm [14,16], is desirable to demonstrate the claimed advantages of our algorithm. It is often not available. Therefore, we use four "high-amplitude" and four "low-amplitude" gene sets identified in [17] as ground truth to evaluate the ability of our algorithm to recover them using subsets of a variety of lengths. The high and low amplitude genes used in this example are well-studied genes in the cell cycle, and many of them are co-regulated by a number of signaling pathways [17,18]. We then use incomplete prior knowledge supplied by our collaborator and apply our algorithm to predict new WNT and NOTCH pathway genes in the somitogenesis process.

### Recovering low and high amplitude gene sets using incomplete prior knowledge

As a proof of concept, we first analyzed a cell cycle data set originally reported in [17]. The data set consists of whole yeast genome expression profiles interrogated over two full cell cycles (20 evenly spaced time points) synchronized by elutriation. We considered the same 308 genes as in the paper derived using Fourier transform. In each of the four gene sets, genes were further classified into high-amplitude and low-amplitude groups according to magnitude of variation. The processed data are available from the authors' website at [19].

We treated the high-amplitude genes and low-amplitude genes in each gene set as "complete", as assumed in classical GSEA analysis. We sampled subsets of increasing sizes from 5 to complete (e.g. 40) with a step size of 5. In each step experiment, we generated 500 subsets of the same size (with replicates), and for each subset we applied our algorithm to demonstrate its ability to recover the full gene set using the hypergeometric test explained in method section. The *P*-values of the tests were used as a measure for such an ability. For visualization convenience, the *P*-values were negatively log-transformed and higher value corresponds to better recovery of the complete gene set.

**Figure 2:**
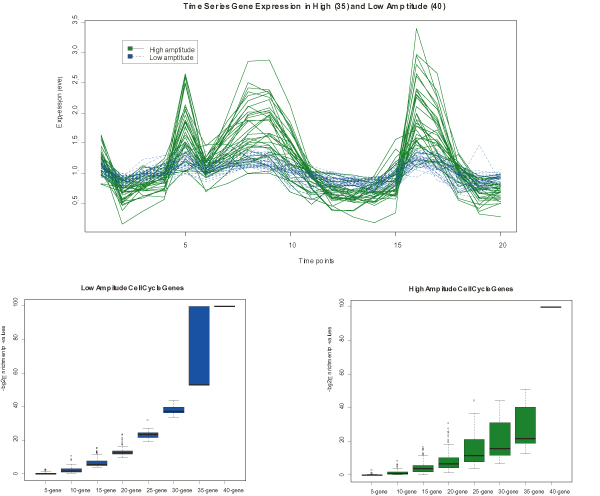
**Demonstration of the claimed advantages of our algorithm using the "ground truth" reported in **[17]. (a) Plots of expression profiles of high-amplitude and low-amplitude gene sets. (b) Evaluating the capability of our algorithm to recover a complete low-amplitude gene set. The gene shaving shaving algorithm [4] fails in this case because it exclusively works with the leading PC. X-axis represents the increasing sizes of the subsets, and Y-axis represents the -*log2P *of the enrichment, indicating increased capacity of recovering a complete gene set. (c) Evaluating the capability of gene shaving algorithm [4] to recover a complete high-amplitude gene set.

The high-amplitude and low-amplitude complete gene sets were plotted in Figure 2a (upper panel of Figure 2). In both Fig 2b (lower left panel of Figure 2) and Fig 2c (low right panel of Figure 2), the ability of recovering the complete gene set (ground truth) was plotted against the increasing subset size respectively. The observed monotonic increase indicates that the larger the subsets (prior knowledge) are, the more capable of recovering the complete gene set. It is worth mentioning that Figure 2b demonstrates the capability of our algorithm to recover low-amplitude gene set, and Figure 2c demonstrates the capability of the gene shaving algorithm [4] to recover high-amplitude gene set.

Our algorithm can be viewed as an generalization of the gene shaving algorithm. Gene shaving algorithm exclusively works with the leading PC. Therefore, it is only capable of identifying high-amplitude signaling pathways. Our algorithm adaptively works with the PC that is most enriched by prior knowledge. Therefore, it is capable of identifying either high-amplitude or low-amplitude signaling pathways wherever prior knowledge is available. Comparing Figure 2b to Figure 2c more closely, it is evident that our algorithm recovers low-amplitude gene sets even better than gene shaving algorithm recovers high-amplitude ones. This is demonstrated by uniformly larger mean values and overall smaller variance on the vertical axis. The results of analyzing other complete gene sets of appropriate size lead to the same conclusion (see additional file 1). The proof-of-concept analysis provided compelling evidence that our algorithm is particularly suitable for identifying sets of tightly regulated genes with modest variation.

### Predicting WNT and NOTCH pathway genes using prior knowledge

#### Microarray data and prior knowledge

We then proceed to re-analyze microarray data originally reported in Dequeant et al [20] to predict genes in WNT and NOTCH pathways. In this experiment, the genome-wide gene expression was interrogated over 17 developmental stages using Affymetrix GeneChip 430A. Using the Lomb-Scargle periodogram [21] the top 687 genes were used for gene clustering so that all prior knowledge genes are included. Microrarray data are available at ArrayExpress at [22].

Prior knowledge corresponds to a list of experimentally validated cyclic genes regulated by the segmentation clock, a molecular oscillator acting during somitogenesis [20]. The segmentation clock is a set of periodic processes linked to the formation of the vertebrate embryo segments (somites) that give rise to the segments in the adult body plan of a vertebrate animal. Malfunction of cyclic genes are the direct cause of many developmental diseases, such as Noonan syndrome and truncated tail [20]. Therefore, predicted cyclic genes are potential human disease genes. In particular, we have incomplete sets of 11 genes in the WNT pathway, and 9 genes in the NOTCH pathway as our prior knowledge. Our objective is to predict more WNT and NOTCH genes using prior knowledge, microarray data and our proposed algorithm.

#### Finding the most enriched PC using prior knowledge

**Figure 3:**
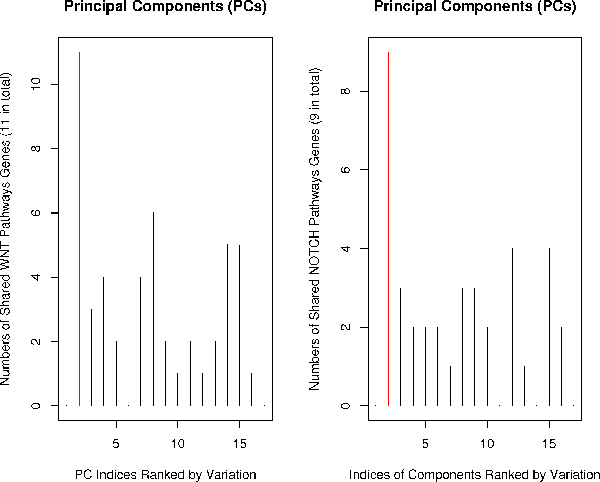
**SVD analysis without splitting negative and positive PC's**. WNT and NOTCH genes are maximally enriched (P-value: E-06) in the second PC (red lines), not the leading PC.

**Figure 4:**
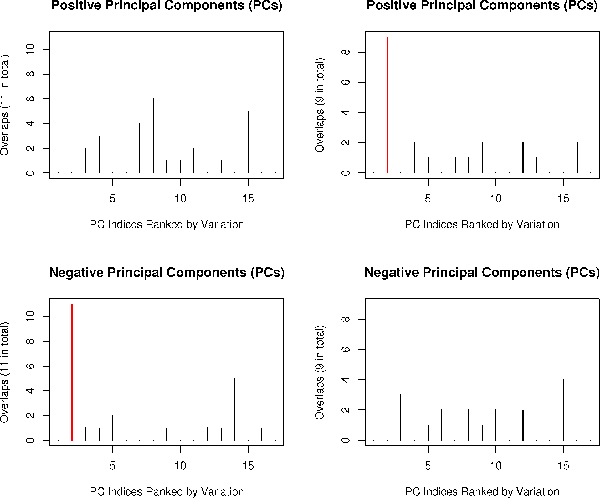
**SVD analysis with splitting negative and positive PC's**. Further, WNT and NOTCH genes are maximally enriched (P-value: E-10) in the second negative PC and the second positive PC (red lines), and the level of enrichment is dramatically increased because the sizes of negative and positive PC's decrease.

In each iteration of our algorithm, we search for the PC that is most enriched by known WNT and NOTCH genes. We filtered the gene coefficients in each PC using the cutoff and tested enrichment of known pathway genes using the hypergeometric test (see method section). Figure 3 shows what happened in the first iteration where all 11 known WNT genes and all 9 known NOTCH genes are included in the second PC (enrichment level is *E *- 06). After separating positive and negative PC's, in Figure 4, all known WNT genes are included in the second negative PC and all known NOTCH genes are included in the second positive PC (enrichment level is *E *- 10). The marked increase of P-value reveals that separating positive PC from negative PC is a key to better enrichment of prior knowledge. The fact that prior knowledge is mostly enriched in PC's other than the leading one indicates that the gene expression in the NOTCH and WNT pathways show only modest and concordant changes. The enrichment of prior knowledge in the gene cluster could be further improved as our algorithm iterates. In the next section, we present results of generating the "best" WNT and NOTCH clusters in which enrichment of prior knowledge is optimized.

#### Comparing our semi-supervised algorithms with the gene shaving algorithm

**Figure 5:**
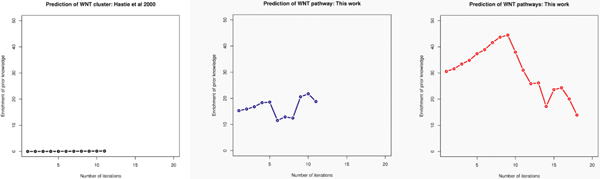
**Algorithm comparisons**. Horizontal axis represents the number of iterations in both upper or lower panels. The vertical axis of the upper panel corresponds to the -*log2P*-value of the enrichment of prior knowledge. The vertical axis of the lower panel corresponds to the number of genes in the cluster (upper) and size of the cluster (lower). (a) The performance of the original gene shaving algorithm gauged by prior knowledge enrichment over iterations [4]. (b) The performance of our semi-supervised gene shaving algorithm without splitting positive and negative PC's. (c) The performance of our semi-supervised gene shaving algorithm with splitting positive and negative PC's.

We aim to show that our semi-supervised algorithm is uniquely able to identify low variation signaling pathway genes but not the gene shaving algorithm. For predicting WNT cluster, our algorithm terminates after 18 iterations, and for predicting NOTCH cluster, it terminates after 20 iterations. We then traced back to retrieve the optimized clusters. Both WNT and NOTCH clusters were retrieved at the 9th iteration that prior knowledge is most enriched, and were smallest clusters containing all prior knowledge genes (Figure 5c). From Figure 5a, the original gene shaving algorithm [4] apparently failed in this case demonstrated by no enrichment of prior knowledge at all. The reason is, as discussed before, that WNT and NOTCH pathway genes are concordantly regulated in modest magnitude while gene shaving algorithm only works with the leading PC. Figures 5b and 5c present the prior knowledge enrichment achieved by two variants of our semi-supervised algorithm: with or without separating positive PC's from negative PC's. It is evident that splitting PC's gives rise to better clustering performance.

**Figure 6:**
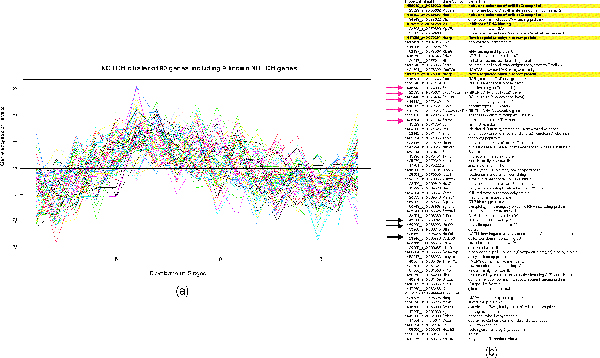
**The predicted NOTCH cluster**. Highlighted genes are prior knowledge. Genes that are pointed by red arrows correspond to experimentally validated NOTCH genes, and genes pointed by blue arrows correspond to potentially interesting genes by expert opinion and literature search. The whole list of prior knowledge and prediction are available in supplemental tables.

The left panel of Figure 6 plots gene expression profiles of the predicted NOTCH cluster, and right panel displays the annotation of those genes. Genes in the shaded areas are from our prior knowledge [20], and genes that are pointed by red arrows indicate the genes are experimentally validated to be positive, and genes pointed by blue arrows indicate the genes are potentially relevant through literature search. Note that the two pathways are far less from well understood, and therefore, many predicted genes, although not currently supported by experimental evidence, are likely to be validated later.

To make our prediction useful for improving current understanding of the mechanisms of WNT and NOTCH pathways in somatogenesis, we performed analysis to infer what kinds of biological functions (defined by Gene Ontology, GO) are most enriched in the pathways, and what kind of transcription factors (inferred through ChIP-chip experiments) are most likely to be involved in regulating the two pathways. Table 1 presents the results of abovementioned enrichment analysis. The analysis was done through the web-server of the Segal lab: [23]. In table 1, results appear to be meaningful since many significantly enriched GO terms (column 3) are related to embryonic development, and both enriched transcription factors (column 4): MyoG and MyoD are closely related to cell differentiation [24,25]. In particular, Myod and Myog have distinct regulatory roles at a similar set of target genes. The role of Myog in mediating terminal differentiation is partially to enhance expression of a subset of genes previously turned on by Myod [25].

**Table 1:** Biological function enrichment analysis and transcription factor association analysis.[[Bibr B23]]

Gene Set	Size	GO Annotation	Transcription Factors
WNT	45	embryonic development (1.13E-04)	MyoG_Myotubes (9.47E-03) [24]
		cytosol (9.15E-06)	MyoD_Growing cells (1.99E-05) [24]
		cytosolic part (4.48E-08)	
		iron ion binding (3.92E-06)	
		tube development (3.86E-04)	
		branching morphogenesis of a tube (9.88E-06)	
		tube morphogenesis (7.26E-05)	
		patterning of blood vessels (3.57E-05)	
		embryonic pattern specification (1.11E-04)	
		oxygen binding (6.89E-14)	
		gas transport (4.60E-14)	
		hemoglobin complex (1.12E-14)	

NOTCH	36	developmental maturation (3.86E-04)	MyoG_Myotubes (9.47E-03) [24]
		negative regulation of cell differentiation (3.01E-04)	MyoD_Growing cells (1.99E-05) [24]
		ectoderm development (1.91E-05)	
		cell maturation (1.94E-04)	
		tissue morphogenesis (1.12E-05)	
		epidermis morphogenesis (2.00E-06)	
		hair cell differentiation (5.26E-06)	
		mechanoreceptor differentiation (7.56E-06)	
		negative regulation of neuron differentiation (3.49E-06)	
		regulation of neuron differentiation (3.93E-05)	
		cell fate determination (9.65E-06)	
		auditory receptor cell fate commitment (3.78E-08)	

The third column contains biological functions significantly enriched in WNT and NOTCH pathways, and the fourth column contains transcription factors significantly associated with WNT and NOTCH pathways. The analysis was done through the web-server of the Segal lab: [23]

#### Stability of clusters against perturbation of prior knowledge

**Figure 7:**
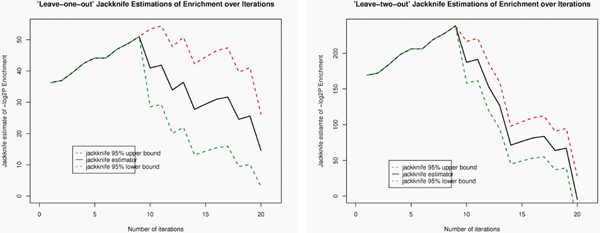
**Leave-one-out and Leave-two-out Jackknife estimations and confidence intervals of the enrichment**. (a) Accessing cluster sensitivity to perturbation of prior knowledge using leave-one-out approach (b) Accessing cluster sensitivity to perturbation of prior knowledge using leave-two-out approach.

Our approach predicts new pathway genes based on the available prior knowledge, therefore, it is critical to investigate the sensitivity of our prediction to a modest perturbation of prior knowledge. Since in this data set we don't know such ground truth as we did in the cell cycle data analysis, we performed sensitivity analysis using leave-one-out and leave-two-out jackknife approaches, see method section for technical details. Narrower Jackknife confidence interval of the enrichment indicates better stability of our enrichment estimation against perturbation of prior knowledge. In Figure 7a where the leave-one-out approach was applied, the estimation of enrichment is perfectly stable (zero variance) and increases until the ninth iteration. Recall that we traced back and retrieved the "best" NOTCH gene cluster right in the ninth iteration. This translates into the fact that our cluster analysis is very robust against moderate perturbation of prior knowledge. In Figure 7b where the leave-two-out approach was used follows a similar trend but with better stability (a narrower confidence interval). This is due to the fact that there are a larger number of Jackknife samples available in leave-two-out approach.

## Discussion

With exception of a few recent works [26-28], most clustering algorithms these days are non-supervised in the sense that prior knowledge is not properly utilized to guide the learning process. Instead prior knowledge is often used in the post-learning phase in that researchers predict functions of unknown genes based on genes of known functions lying in the same cluster. The traditional gene shaving method focuses on the leading PC that accounts for most of variation in the data. On one hand, it is useful in discovering high variation pathway genes [4,29], on the other hand, it tends to overlook essential pathway genes that have modest expression variation. We hypothesized that highly concerted expression behavior of these genes, albeit modest in variation, may help shape its pattern out of the noisy microarray data using appropriate analysis techniques, i.e., SVD.

The main contribution of this work is that we proposed an optimization algorithm combining the strengths of gene set based analysis and iterative gene selection. The iterative fashion inspired from the gene shaving algorithm allows distilling desired gene cluster using prior knowledge, while the latter enables us to discover gene clusters of modest and concerted expression change. The PC's that define gene clusters group a series of tightly regulated genes ranked by variance over samples. The orthogonality as specified in SVD analysis indicates those gene clusters of different variation were regulated by orthogonal defined or undefined genomic influences (Table 1 of [11]).

Our method is particularly suitable for identifying gene clusters with modest and concerted expression change, therefore it is not limited to identify periodically expressed gene clusters. When there is no prior knowledge available, the optimization process can be done through optimizing the enrichment of interesting Gene Ontology (GO) vocabulary, for example, somitogenesis [GO:0001756]. The technique for testing enrichment of GO term is very similar to that was used here, also see review in [30]. A recursive dendrogram can be constructed as a foundation to generate overlapping gene clusters, from which the optimal clusters can be identified and retrieved according to the enrichment of the interesting GO term(s) [3].

## Conclusion

Our algorithm represents one of the first clustering algorithms that is particularly designed to identify signaling pathways of low and concordant gene expression variation. The discriminating power is achieved by manufacturing a principal component enriched by the prior knowledge.

## Methods

### Singular Value Decomposition

Assume the gene expression data is in the matrix format *X*_*p *× *n*_, where rows (*p*) correspond to genes and columns (*n*) correspond to conditions under which gene expression abundance were interrogated. Singular value decomposition (SVD) of the rectangular matrix *X *can be expressed as follows:

(1) $$X_{p\times n}=U_{p\times n}S_{n\times n}V_{n\times n}^{T},$$

where *U*_*p *× *n *_is the gene coefficient, and *U*_*ij *_is the contribution of *i*_*th *_, *i *= 1, ..., *p*, gene to the *j*_*th*_, *j *= 1, ..., *n*, PC. If we correspond each *U*_*j *_to a genomic influence *j*, then *U*_*ij *_defines how much the gene *i *is regulated by the genomic influence *j*. *S*_*n *× *n *_is the singular value matrix, where the diagonal contains list of singular values, and the magnitude of singular values corresponds to percentage of variation explained by each PC. $V_{n\times n}^{T}$ stores PC's [9,10]. We then separated positive PC's from negative PC's according to the signs of entries in *U*_*p *× *n*_, i.e.,

(2) $$X_{p\times n}=X_{p\times n}^{+}+X_{p\times n}^{-}.$$

Refer to supplemental figure 1 for a schematic illustration of the procedure. As shown in later data analysis examples, the separation operation is the key to enhance the prior knowledge enrichment level and to differentiate between antiphased WNT and NOTCH clusters.

### Testing gene coefficients

Smaller fraction numbers of *U*_*ij *_may indicate the contribution of *i*_*th *_gene to *j*_*th *_PC is negligible. We used a cut-off value that was originally used in [10] to test the vanishing of *U*_*ij *_(similar to a 3*σ *statistical significance):

(3) $$U_{ij}=\{\begin{array}{rr} U_{ij} & \text{for }|U_{ij}|>\frac{p}{\sqrt{n}},\\ 0 & \text{for }|U_{ij}|\leq \frac{p}{\sqrt{n}}. \end{array}$$

Each element in $X_{p\times n}^{+}$ and $X_{p\times n}^{-}$ is compared to the value $\frac{p}{\sqrt{n}}$, where *n *is the number of genes and *p *is a weight factor whose recommended value is 3. If the magnitude of the element in $X_{p\times n}^{+}$ and $X_{p\times n}^{-}$ is greater than $\frac{p}{\sqrt{n}}$, the corresponding gene is determined to contribute significantly to the PC's. Alternatively the list of genes that are significantly up-regulated or down-regulated by the underlying genomic influence corresponding to each PC.

### Enrichment test

For each PC *j*, suppose there is a gene set *K *of *k *genes that *U*_*ij *_is not 0, and for a biological pathway, suppose there is a prior knowledge gene set *M *of *m *genes in known in the pathway. Also assume there are *n *genes NOT in the pathway, and *x *is the number of common genes shared by *K *and *M*. The probability of observing exactly *x *common genes is:

(4) $$P(X=x)=\frac{\left(\begin{array}{c} m\\ x \end{array}\right)\left(\begin{array}{c} n\\ k-x \end{array}\right)}{\left(\begin{array}{c} m+n\\ k \end{array}\right)}.$$

In order to estimate the probability of observing *x *common genes or more is purely due to chance, we test the following one-sided hypothesis:

(5) $$H_{0}:O_{1}=O_{2}\text{ versus }O_{1}\geq O_{2},$$

where $O_{1}$ is a parameter corresponding to the probability of genes in the prior knowledge belonging to the PC, and $O_{2}$ is a parameter corresponding to the probability of genes not in prior knowledge belonging to the PC. Under *H*_0_, the test statistic *x *follows a hypergeometric distribution with known parameters *m*, *n *and *k*.

The *P*-value is then defined as the probability of observing *x *or more overlaps given *H*_0 _is true. Therefore, it is calculated as follows:

(6) $$\begin{array}{lll} P_{V} & = & P(X\leq x)\\ & = & 1-P(X<x)\\ & = & 1-\sum_{o=1}^{x-1}P(X=o)\\ & = & 1-\sum_{o=1}^{x-1}\frac{\left(\begin{array}{c} m\\ o \end{array}\right)\left(\begin{array}{c} n\\ k-o \end{array}\right)}{\left(\begin{array}{c} m+n\\ k \end{array}\right)} \end{array}$$

### Semi-supervised gene shaving algorithm

1: Start with the centered data matrix *X *that each row has zero mean

2: **while **TRUE **do**

3:    Singular value decomposition $X_{p\times n}=U_{p\times n}^{+}S_{n\times n}V_{n\times n}^{T}+U_{p\times n}^{-}S_{n\times n}V_{n\times n}^{T}$

4:    **for all **column of $U_{p\times n}^{+}$ and $U_{p\times n}^{-}$**do**

5:       if column elements are greater than a cut-off **then**

6:          NO change

7:       **else**

8:          Set to 0

9:       **end if**

10:       **end for**

11:       **for all **Gene sets correspond to each columns **do**

12:          Test enrichment of prior knowledge in each gene set

13:       **end for**

14:       **if **Two or more columns that are most enriched with prior knowledge exist **then**

15:          Break

16:       **else**

17:          Retrieve the best PC that are most enriched by prior knowledge

18:       **end if**

19:          Sort genes according to absolute correlation with the best PC

20:          Discard *α*% least correlated genes (*α *= 10% followed from [4])

21:          Assign the reduced data matrix to *X*

22: **end while**

23: Trace-back to retrieve the best gene cluster

As shown in the above Algorithm and Figure 1, the algorithm iterates until there are two or more most enriched PC's coexisting as defined by prior knowledge. The iterations stop here since we don't yet know a good way to further reduce the size of the cluster. Inconsiderate reduction might cause a loss of important genes. There are two ways of tracing back to retrieve the best gene cluster. One is to find the smallest cluster containing all prior knowledge, another is to find the cluster in which the enrichment of prior knowledge optimized. We chose the latter because it does not rely on the assumption that all prior knowledge need to be accurate. In fact, each gene coefficient can be used to measure the relative importance of genes in forming the cluster pattern. Genes in prior knowledge that help shaping out patterns receive higher weight, otherwise receive lower weight.

### Stability analysis of gene clusters – a jackknife approach

Jackknife approach, e.g. "leave-one-out", is a resampling approach that is frequently used to access the stability of an estimator such as enrichment studied here. Suppose we wish to estimate enrichment parameter (*η*) as a complicated statistic (*T*) of *n *genes in prior knowledge as well as D,

(7) $$\hat{\eta }=T(g_{1},g_{2},...,g_{i-1},g_{i},g_{i+1},...,g_{n},D).$$

Let *j*th partial estimate of *η *be given by the estimate computed with gene *i *removed,

(8) $${\hat{\eta }}_{j}=T(g_{1},g_{2},...,g_{i-1},g_{i+1},...,g_{n},D).$$

The jackknife estimate of *η *is given by the average of the pseudovalues [31],

(9) $$\eta^{*}=\frac{1}{\eta }\sum_{i=1}^{n}\left(n\hat{\eta }-(n-1){\hat{\eta }}_{j}\right).$$

An approximate sampling error for ${\hat{\eta }}^{*}$ can be obtained as the following [31]:

(10) $$Var(\eta^{*})=\frac{Var(\eta_{j}^{*})}{n}=\frac{\sum_{j=1}^{n}{\left(\eta_{j}^{*}-\eta^{*}\right)}^{2}}{n(n-1)}.$$

Likewise, an approximate (1 - *α*)% confidence interval is given by [31],

(11) $$\eta^{*}\pm t_{\alpha /2,n-1}\sqrt{\frac{\sum_{j=1}^{n}{\left(\eta_{j}^{*}-\eta^{*}\right)}^{2}}{n(n-1)}},$$

where *t*_*α*/2, *n*-1 _satisfies *Pr*(*t*_*n *_≥ *t*_*α*/2, *n*-1_) = *α*, with *t*_*n *_denoting a *t*-distributed random variable with *n *degree of freedom.

## Competing interests

The author declares that they have no competing interests.

## Author's contributions

DZ conceived and designed the method, analyzed data and drafted the manuscript.

## Supplementary Material

Additional file 1 Supplemental figures. Click here for file
