# Supplementary material for: Semi-supervised gene shaving method for predicting low variation biological pathways from genome-wide data
Source: BMC Bioinformatics. 2009 Jan 30;10(Suppl 1):S54. doi: 10.1186/1471-2105-10-S1-S54 (PMC2648790; doi:10.1186/1471-2105-10-S1-S54)
Supplement: Additional file 1 — Supplemental figures. [file 1471-2105-10-S1-S54-S1.doc]

Fig. S1. Schematic overview of the proposed algorithm

Fig. S2. Demonstration of the claimed advantages of our algorithm using the “ground truth” reported in Rustici *et al*., 2004. (a) Plots of expression profiles of high-amplitude (in second gene set) and low-amplitude gene sets (in fourth gene set). (b) Evaluating the capability of our algorithm to recover a complete low-amplitude gene set. X-axis represents the increasing sizes of the subsets, and Y-axis represents the -*log*2*P* of the enrichment, indicating increased capacity of recovering a complete gene set. (c) Evaluating the capability of our algorithm to recover a complete high-amplitude gene set.

Fig. S3. Algorithms comparison in identifying WNT cluster. Three columns correspond to three clustering algorithms. The first one (left-hand-side) is the original gene shaving algorithm (Hastie *et al*. (2000)). The second and third ones are our new algorithms (without and with splitting positive and negative PC’s). Horizontal axis represents the number of iterations in both upper and lower panels. The vertical axis of the upper panel corresponds to the *¡log*2*P*- value of the enrichment of prior knowledge. The vertical axis of the lower panel corresponds to the number of genes in the cluster (upper) and size of

the cluster (lower).

Fig.S4. The predicted best NOTCH cluster. Highlighted genes are prior knowledge.
